# Domain-Dependent Performance of Human Experts and AI Systems in Pediatric Menu Evaluation

**DOI:** 10.3390/nu18183106

**Published:** 2026-09-21

**Authors:** Roxana Maria Martin-Hadmaș, Rebeca Sovea, Diana Pol, George Mihăiță Gavra, Monica Tarcea, Adriana Neghirlă, Ștefan Adrian Martin

**Affiliations:** 1 Department of Community Nutrition and Food Safety, “George Emil Palade” University of Medicine, Pharmacy, Science and Technology of Târgu Mures, Gheorghe Marinescu 38, 540139 Targu Mures, Romania; roxana.hadmas@umfst.ro (R.M.M.-H.); sovea.rebeca-eliza.25@stud.umfst.ro (R.S.); pol.diana-florina.25@stud.umfst.ro (D.P.); monica.tarcea@umfst.ro (M.T.); 2 Center for Advanced Medical and Pharmaceutical Research, George Emil Palade University of Medicine, Pharmacy, Science, and Technology of Targu Mures, 540139 Targu Mures, Romania; george.gavra@umfst.ro; 3 Mures School Medicine Association, 540233 Targu Mures, Romania; adi_neghirla@yahoo.com

**Keywords:** artificial intelligence, pediatric nutrition, dietary assessment, menu evaluation, large language models, energy estimation, nutrition professionals, clinical decision support

## Abstract

Background/Objectives: Artificial intelligence (AI) tools are increasingly used for dietary assessment, but their reliability in pediatric nutrition remains uncertain. This study compared AI systems and human evaluators in pediatric menu assessment against expert-defined reference ratings. Methods: This observational cross-sectional study used anonymized dietary and clinical information from three healthy pediatric cases. A multidisciplinary panel of pediatric nutrition specialists established standard evaluations. Assessments were completed by 84 AI evaluations, 116 nutrition specialists, 56 pediatric-focused physicians, and 88 physicians from other specialties. Outcomes included absolute error in total energy estimation, deviations in portion and qualitative ratings, standardized scores, and binary adequacy accuracy. Groups were compared using ANOVA or Kruskal–Wallis tests with post hoc analyses. Results: A total of 344 assessments were included. Energy-estimation error differed between groups (Kruskal–Wallis = 12.85, *p* = 0.0016), but this analysis was based on a limited and highly unbalanced subset of 77 assessments. AI had the largest mean absolute error (260.3 ± 194.3 kcal), followed by nutrition specialists (148.4 ± 81.5 kcal); physicians with other specialties had the lowest error (60.4 ± 53.3 kcal); these subgroup comparisons should be interpreted cautiously because of the small and unequal numbers of available energy estimates. AI showed greater portion-rating deviation (0.488; 95% CI: 0.38–0.60) than nutrition specialists (0.310; 95% CI: 0.20–0.42; *p* = 0.0019, q = 0.0059) and pediatric-focused physicians (0.286; 95% CI: 0.16–0.41; *p* = 0.0181, q = 0.019). Conversely, AI showed the lowest deviations for variety (0.37 ± 0.49) and processing (0.33 ± 0.47), compared with 1.15–1.36 and 1.22–1.57, respectively, among human groups (both *p* = 0.0001). Conclusions: AI may support structured pediatric menu screening for descriptive qualitative features; however, lower precision for energy and portion assessment supports, under these specific conditions, its use as an adjunct, for qualified nutrition professionals.

## 1. Introduction

Nutrition is essential for growth, neurodevelopment, prevention of obesity, and diet-related cardiometabolic disorders. However, routine evaluation of children’s menus in clinical and community settings remains time-consuming and highly dependent on individual professional expertise.

Technological tools for assessing food intake have been developed to improve standardization and reduce respondent burden, but their validity and usability are heterogeneous, and many still require expert interpretation [1]. At the same time, large language models (LLMs) and artificial intelligence-driven chatbots (AI) have become freely and widely accessible, allowing parents to obtain instant diets and health information for their children from general-purpose systems without direct contact with healthcare professionals. Recent studies indicate that parents are increasingly using AI for pediatric medical and nutritional queries and may sometimes trust AI-generated content more than experts’ information, raising concerns about safety and the potential displacement of qualified advice [2,3]. AI dietary assessment tools based on images and mobile interfaces show promising performance in food detection and portion estimation, but nutrient and energy quantification remain challenging [4,5,6,7], particularly when meals contain unknown ingredients, mixed dishes, variable recipes, and imprecisely estimable amounts of added fats, oils, sauces, and cooking losses [8].

Despite the growing interest in AI-supported dietary assessment, the existing literature has largely examined isolated tasks such as food recognition, portion estimation, or nutrient prediction under simplified conditions. Few studies have directly compared AI outputs with those of multiple human evaluator groups in pediatric menu assessment [9]. As a result, it remains unclear whether AI performs differently across quantitative and qualitative dimensions of menu evaluation. Despite promising accuracy in controlled settings, current LLM-based nutrition tools still show important limitations in external validity, with performance shaped by prompt design, language context, and incomplete real-world inputs. In one Polish dietary classification study, three models reached 90.3–94.2% agreement with experts, yet significant model–human differences persisted, and expert oversight remained essential [10].

As a result, the present study was designed to compare the accuracy, and domain-dependent performance of pediatric menu evaluations generated by three human evaluator groups (nutrition specialists, pediatric-focused physicians, and physicians with other specialties) and AI platforms, using expert-defined ratings across energy intake, portion, quantity assessments, and key qualitative attributes. By quantifying deviations from the reference standard and examining binary adequacy indicators and evaluator characteristics among human experts, we aimed to clarify the current role of AI as an adjunct tool for structured pediatric menu screening and nutritional education, rather than as a substitute for multidisciplinary clinical expertise [11].

## 2. Materials and Methods

We conducted an observational, cross-sectional, comparative study in Târgu Mureș, Romania, using anonymized pediatric cases and standardized menu-evaluation instruments. Evaluations were completed online between March and June 2026 via secure survey links distributed to human evaluators and via individual sessions for AI platforms. The study was conducted in accordance with the principles of research ethics and scientific integrity. It was approved by the Ethics Committee of the “George Emil Palade” University of Medicine, Pharmacy, Science, and Technology of Târgu Mureș under document no. 3904/4 March 2026.

The research methodology used anonymized pediatric data derived from three real cases for which the legal guardians provided written informed consent for the clinical and dietary information to be used in research and educational contexts. Participation by human evaluators was voluntary, with electronic informed consent obtained before data collection. No child or evaluator was exposed to physical or psychological risk, and no identifying information was collected in the evaluation forms or AI prompts.

### 2.1. Pediatric Cases and Standard Evaluations

Three pediatric cases—male, 7 years; female, 12 years; female, 3 years—without known chronic disease were selected to represent distinct anthropometric and cardio-metabolic profiles and clearly differentiated dietetic approaches in otherwise healthy children while avoiding extreme scenarios. This choice was intended to minimize the risk that between-group differences in performance would be driven by a single atypical case (e.g., very young age, unusually clinical situation). Each case included information regarding age, sex, weight, height, Body Mass Index (BMI) percentiles, resting metabolic rate, selected cardiometabolic parameters, and a detailed food diary with portion descriptions (food weight), photos, and meal serving program.

A multidisciplinary reference panel comprising three clinical nutrition specialists, two pediatricians, and one school medicine physician established the case-specific expert reference ratings. None of the six panel members participated in the human evaluator sample or in the comparative analyses. For each pediatric case, the panel reviewed the anonymized clinical and anthropometric information, detailed dietary record, food weights and portion descriptions, meal photographs, and meal-serving information. Total energy and nutrient values were calculated using the USDA FoodData Central database and interpreted in relation to current pediatric nutrition guidance [12]. The reference assessment included total daily energy intake, portion-size and quantity categories, binary adequacy indicators, ordinal ratings of variety, processing level, nutritional quality, color, and attractiveness, global menu class, and standardized 1–10 domain scores. The reference ratings were developed through direct multidisciplinary discussion of each case until unanimous consensus was reached. Panel members did not first provide independent ratings, and inter-rater agreement was therefore not calculated before consensus formation. For outcomes with an interpretive component, particularly variety, processing level, nutritional quality, color, and attractiveness, the resulting values represent consensus-based expert reference standards rather than objective biological measurements. The three pediatric cases were intentionally selected as standardized assessment vignettes representing distinct, clinically plausible healthy-child dietary scenarios. They were not analysed as a random sample of pediatric participants and were not intended to provide population-level estimates of child characteristics. Rather, they served as fixed standardized stimuli against which evaluator agreement with case-specific expert reference ratings was assessed.

### 2.2. Human Evaluators

Human participants were specialists and trainees from Romania. They were grouped as: (1) nutrition specialists (*n* = 116), (2) pediatric-focused physicians (family medicine, school medicine, pediatrics, pediatric gastroenterology, etc.; *n* = 56), and (3) physicians with other specialties, without specific pediatric specialization (laboratory medicine, pathology, hygiene, etc.; *n* = 88). Human evaluators were recruited using a multi-channel strategy that included institutional mailing lists from universities and research centers, professional associations in pediatrics and nutrition, as well as targeted e-mails. Recruiting was performed without pre-screening for performance, to simulate a real-world spectrum of professionals involved in pediatric nutrition.

For each evaluator, we recorded professional category, domicile region (N, NE, NV, C, S, SV, SE), primary practice setting (public, private, mixed), years of professional experience, declared level of involvement in nutritional assessment (none, basic, complex), dietary recommendation (none, basic, complex), and collaboration with registered dietitians (frequency/yes–no). We excluded from this study evaluators who operate in another country or who operate in Romania but with medical studies completed in other countries.

### 2.3. AI Evaluators and Prompt

A total of 42 AI platforms were selected to reflect the diversity of AI-based tools and access modes commonly available to the general public and healthcare professionals in Romania at the time of data collection. Each platform was assessed under two distinct configurations, defined by access type (free or paid), model or version where identifiable, and/or relevant operating mode or enabled feature (e.g., standard, reasoning/thinking, deep-research, or web-enabled mode). Thus, the study included 84 distinct platform-configuration assessments. One AI assessment was defined as one completed evaluation generated by one specified platform under one specified access tier, model/version where identifiable, and operating configuration, for one assigned pediatric case vignette. The two configurations associated with the same platform were applied to different pediatric case vignettes. The selection was designed to capture variation across realistically available AI access conditions and configurations, rather than to estimate within-platform test–retest reliability. Each AI assessment received the same pediatric case information and visual/journal dietary data as the human evaluators, together with a standardized prompt requesting: (1) a quantitative evaluation of intake (portions, amounts, balance), (2) a qualitative evaluation of food quality and presentation (variety, processing, color, attractiveness), (3) global class assignment into five predefined categories, (4) five 1–10 ratings (quantitative, qualitative, nutritional, attractive, and color), and (5) a self-reported level of involvement in nutritional assessment and recommendation, classified according to the same predefined categories used for human evaluators.

Eligibility as an AI evaluator required that the selected platform or configuration could receive the standardized case materials in a format compatible with its available interface and could generate a written response to the Romanian-language evaluation prompt. For platforms that did not support direct image upload or the full range of input modalities, the same case-specific clinical information, food diary, food weights, portion descriptions, and meal information were provided in the closest supported text-based format. All AI assessments were required to address the same predefined evaluation domains and response structure.

All AI interactions used the same clear, non-technical language and structure, designed to mimic how a parent or a non-IT-specialized healthcare professional would naturally query an AI assistant about a child’s daily menu. Prompts were phrased in Romanian for all platforms, and no code-like syntax or advanced AI-specific instructions were included to approximate real-world use. Where platforms allowed web browsing or plug-ins, these features were left at default settings, and no external databases were manually provided beyond the case descriptions.

The complete standardized Romanian-language prompt and a faithful English translation are provided in Supplementary Files.

### 2.4. Evaluation Instrument

Human evaluators completed a structured nutritional evaluation report for each case, aligned with the same dimensions as the AI prompt: quantitative assessment, qualitative assessment, global class (1–4 plus “unable to evaluate”), and five standardized 1–10 scores (quantitative, qualitative, nutritional, attractive, color). The instrument provided definitions for score ranges (1–3 poor, 4–6 moderate, 7–8 good, 9–10 excellent) and class descriptors, while explicitly allowing evaluators to use their own tools and calculations, provided that the structure was respected.

Each human evaluator completed one structured evaluation of one randomly assigned pediatric case vignette. Before accessing any case materials, evaluators were presented with three numerically labelled case options and selected one option without being able to view the corresponding case content in advance. Once a case had been selected and accessed, the remaining two case options became unavailable. Thus, case selection was effectively random with respect to case content and was not assigned by the investigators according to evaluator professional category, experience, or other characteristics. No quotas, stratification, or balancing procedure was applied; consequently, the final number of evaluations differed across the three case vignettes. Therefore, no human evaluator contributed repeated assessments, and each human assessment represented one independent evaluator response. The primary analytic unit was the evaluator–case assessment. All outcome measures were calculated as deviation from the expert-defined reference standard for the assigned case; thus, each response was evaluated relative to the appropriate case-specific benchmark rather than against a common child-level outcome. AI systems were evaluated using the same standardized case materials and predefined output structure. The purpose of the analysis was to compare the distribution of evaluator-level agreement with case-specific reference standards across AI and human evaluator groups under standardized assessment conditions, rather than to estimate population-level differences between pediatric patients. If an evaluator did not provide a total daily energy estimate, energy deviation could not be computed, and that evaluator was excluded from analyses involving energy-estimation error but remained eligible for analyses of ordinal and binary outcomes. For binary indicators, binary agreement was calculated as the proportion of completed binary items that matched the expert reference standard. This metric was calculated only when at least two binary items had been completed; otherwise, the binary-agreement score was treated as missing for that evaluator.

### 2.5. Statistical Analysis

Analyses were performed using Microsoft Excel (Microsoft Corporation, Redmond, WA, USA) for data preprocessing and computation of evaluator-level deviation metrics, and GraphPad Prism 9 (LLC/Dotmatics, Boston, MA, USA) for all inferential statistics.

All tests were two-sided, with statistical significance set at α = 0.05. For each evaluator–case pair in the database, we computed three families of deviation metrics relative to the expert standard ratings: (1) the absolute difference between the evaluator’s estimated total daily energy intake and the standard for the corresponding case (MAE_kcal); (2) the absolute difference between the evaluator’s rating and the standard rating on the predefined 3-point scale or class scale (diff) for portion size and each qualitative dimension (variety, processing level, nutritional quality, color, attractiveness); (3) binary agreement (Acc_bin), calculated as the proportion of completed binary indicators that matched the expert reference standard (coded 1/0). The pediatric cases were treated as fixed standardized assessment scenarios, not as independent pediatric participants or repeated clinical observations. Because each human evaluator assessed only one randomly assigned case and outcomes were expressed as deviations from the corresponding expert standard, the inferential analyses compared evaluator-level performance across the predefined assessment scenarios. The analyses were not intended to estimate between-child effects or to generalize case-level variance to the pediatric population.

Between-group differences were tested using ordinary one-way ANOVA for unpaired data when the assumptions of approximate normality and homoscedasticity were reasonably met, and the Kruskal–Wallis test when these assumptions were violated. When the global F-test (ANOVA) or H-test (Kruskal–Wallis) indicated significant differences between groups (*p* < 0.05), we performed post hoc multiple comparisons. For parametric ANOVA, we used Tukey’s test to compare all pairs of groups while controlling the family-wise error rate. For nonparametric Kruskal–Wallis analyses, pairwise comparisons were performed using Dunn’s procedure. False discovery rate was controlled across the six pairwise group comparisons generated within each outcome using the two-stage linear step-up procedure of Benjamini, Krieger, and Yekutieli. Nominal two-sided *p* values and false-discovery-rate-adjusted q values are reported for post hoc comparisons. Given that only three deliberately selected standardized cases were included, no case-level random-effects model was fitted, because the number of clusters was insufficient for stable estimation of a between-case variance component. Accordingly, results are interpreted as comparative evaluator performance within the three standardized pediatric scenarios.

Associations between energy-estimation error, evaluator characteristics, and deviation outcomes were explored using Spearman’s rank correlation because several variables were ordinal, discrete, bounded, and/or non-normally distributed. Correlations were based on complete observations for each variable pair and were considered exploratory.

To investigate whether evaluator characteristics were associated with selected agreement-related deviation outcomes among human evaluators, we fitted exploratory multiple linear regression models. The selected deviation outcomes were used as continuous dependent variables, whereas professional category, domicile region, practice setting, years of professional experience, level of nutritional-assessment involvement, level of dietary-recommendation involvement, and collaboration with a registered dietitian were entered as predictors. Categorical predictors were entered as nominal variables using indicator coding, with prespecified reference levels (e.g., public practice setting, central region, and minimal involvement in assessment/recommendation).

MAE kcal was not included in the multivariable regression models because total-energy estimates were available for only a small and uneven subset of human evaluators. It was examined descriptively and in exploratory bivariate correlation analyses only. Regression analyses were restricted to human evaluators because region, professional experience, practice setting, involvement, and collaboration variables were not applicable to AI systems.

Several deviation metrics were derived from limited ordinal or bounded scales. For the exploratory multivariable models, these outcomes were treated as approximately continuous to enable simultaneous assessment of evaluator characteristics and to yield interpretable regression coefficients. This approximation may not fully capture the distributional properties of outcomes with few possible values or a high frequency of zero deviation; accordingly, regression findings are interpreted as exploratory. Residual distributions, residual-versus-fitted plots, and multicollinearity diagnostics were examined in GraphPad Prism.

## 3. Results

A total of 344 standardized assessments were included in the between-group analyses: 84 AI assessments, 116 nutrition specialists, 56 pediatric-focused physicians, and 88 physicians with other specialties. For qualitative outcomes, complete data were available for 336 assessments.

### 3.1. Energy Estimation Results

Among evaluations that included a total energy estimate, MAE_kcal differed significantly between evaluator groups (Kruskal–Wallis statistic = 12.85, *p* = 0.0016). This analysis was based on only 77 assessments with an available energy estimate and was markedly unbalanced across groups (AI, *n* = 61; nutrition specialists, *n* = 8; pediatric-focused physicians, *n* = 0; physicians with other specialties, *n* = 8; Supplementary Table S2). AI assessments showed the largest mean energy-estimation error (260.3 ± 194.3 kcal; median 225 kcal), followed by nutrition specialists (148.4 ± 81.5 kcal; median 120 kcal), while physicians with other specialties had the smallest error (60.4 ± 53.3 kcal; median 46.5 kcal). Post hoc comparisons indicated that the only between-group comparison that remained significant after multiplicity correction was between AI and physicians with other specialties (unadjusted *p* = 0.0005; q = 0.001). Given the limited availability of kcal estimates, this result should be considered exploratory.

When the sample was stratified according to whether a total kcal estimate had been provided, evaluators who reported kcal values were generally closer to the reference standard in several non-energetic domains. Compared with those who did not report kcal values, the kcal-calculation group had lower mean deviations for variety (0.854 vs. 1.341, *p* < 0.0001), processing (0.596 vs. 1.295, *p* < 0.0001), nutritional quality (0.281 vs. 0.605, *p* = 0.0001), attractiveness (0.921 vs. 1.551, *p* < 0.0001), global evaluation (2.247 vs. 4.699, *p* < 0.0001), and color score (3.146 vs. 7.061, *p* < 0.0001), and also showed higher binary accuracy (0.646 vs. 0.375, *p* < 0.0001). In contrast, color deviation, as well as the standardized quantitative, qualitative, nutritional, and attractiveness ratings, did not differ significantly according to kcal-calculation status.

### 3.2. Ordinal Scale Results

For portion ratings, the overall between-group difference was significant (Kruskal–Wallis statistic = 13.92, *p* = 0.003). Mean deviations from the standard were 0.488 for AI (95% CI 0.38–0.60), 0.31 for nutrition specialists (95% CI 0.20–0.42), 0.286 for pediatric-focused physicians (95% CI 0.16–0.41), and 0.455 for physicians with other specialties (CI 0.35–0.56). Post hoc test corrections showed significantly higher deviations in AI compared with nutrition specialists (q = 0.0059, *p* = 0.0019) and pediatric-focused physicians (q = 0.019, *p* = 0.0181), and significantly higher deviations in physicians with other specialties compared with nutrition specialists (q = 0.0118, *p* = 0.0075) and pediatric-focused physicians (q = 0.0366, *p* = 0.0465).

The clearest between-group differences were observed for the qualitative ordinal outcomes (Table 1). AI showed the lowest deviations for both variety and processing, with mean values of 0.37 ± 0.49 and 0.33 ± 0.47, respectively, whereas the human evaluator groups ranged from 1.15 to 1.36 for variety and from 1.22 to 1.57 for processing. By contrast, nutrition specialists had the highest deviation for nutritional quality ratings (1.00 ± 1.02), while the other groups showed lower values ranging from 0.27 to 0.33. No statistically significant between-group difference was found for color ratings (Table 1).

A similar domain-dependent pattern was observed for the standardized 1–10 ratings presented in Table 2. Pediatric-focused physicians had the lowest deviation for the qualitative standardized score (0.29 ± 0.46; median 0), while nutrition specialists and physicians with other specialties had the largest deviations (2.07 ± 2.03 and 2.00 ± 2.64, respectively). For the standardized nutritional score, pediatric-focused physicians showed the lowest mean deviation (1.29 ± 2.00), whereas nutrition specialists had the highest deviation (1.86 ± 1.26). For attractiveness ratings, pediatric-focused physicians and physicians with other specialties remained closest to the standard, while nutrition specialists showed the greatest deviation (Table 2).

### 3.3. Energy Estimation and Ordinal Ratings

The exploratory associations between energy-estimation error, evaluator characteristics, and deviation from the case-specific expert reference standard are shown in Table 3. Higher MAE kcal was positively associated with greater deviation in processing ratings (r = 0.402, 95% CI 0.205 to 0.567, *p* < 0.0001), variety ratings (r = 0.559, 95% CI 0.391 to 0.690, *p* < 0.0001), and negatively with the global evaluation (r = −0.499, 95% CI −0.644 to −0.319, *p* < 0.0001), and standardized color ratings (r = −0.365, 95% CI −0.537 to −0.163, *p* = 0.0004). No association was observed between MAE kcal and binary agreement (r = 0.003, 95% CI −0.211 to 0.217, *p* = 0.976).

In the human-evaluator subset, greater professional experience was associated with lower deviation for variety (r = −0.302, 95% CI −0.386 to −0.214, *p* < 0.0001), and lower deviation for attractiveness (r = −0.253, 95% CI −0.338 to −0.163, *p* < 0.0001). No significant correlations were observed between professional experience and deviation in portion or quantity ratings. Collaboration with a registered dietitian was weakly positively associated with attractiveness deviation (r = 0.125, 95% CI 0.032 to 0.216, *p* = 0.0071).

### 3.4. Human Evaluator Multivariable Regression Analyses

Exploratory multivariable linear regression analyses restricted to human evaluators are summarized in Table 4. The models included professional category, years of experience, domicile region, practice setting, level of nutritional assessment involvement, level of dietary recommendation involvement, and collaboration with a registered dietitian. The models explained between 35.5% and 84.7% of the observed variance across the examined deviation outcomes; however, because several outcomes were bounded or ordinal-derived and the analyses involved multiple predictors, these findings are interpreted as exploratory. Full coefficients, 95% confidence intervals, *p* values, and multicollinearity diagnostics are available in Supplementary Table S3.

All models were statistically significant. Model significance ranged from F (13, 238) = 10.08 for portion deviation to F (13, 238) = 101.4 for nutritional standardized deviation, with all *p* < 0.0001 (Table 4).

Selected adjusted coefficients for the two main quantitative outcomes are detailed in Table 5. Using nutrition specialists as the reference category, both pediatric-focused physicians and physicians with other specialties had significantly higher adjusted deviations for portion and quantity ratings. Experience was independently associated with lower deviation for quantity ratings, but not for portion ratings (Table 5).

## 4. Discussion

The present study compared pediatric menu evaluations generated by multiple AI systems and three groups of human evaluators against expert-defined standard ratings across quantitative and qualitative domains. The main finding suggests that performance varied strongly by assessment domain, rather than showing consistent superiority of either AI or human assessors. Under these structured conditions, AI showed smaller deviations for descriptive attributes such as menu variety and processing level, whereas energy estimation error remained higher and performance in portion- and quantity-related judgments was less consistent than that of nutrition specialists.

The results are broadly consistent with the current literature. Recent reviews suggest that AI applications in nutrition are advancing fast, with particularly strong development in dietary assessment, food recognition, and structured classification tasks, but with more limited evidence for reliable nutrient quantification, individualized planning, and broader clinical decision support [7,13]. Studies on AI-assisted dietary assessment indicate that while AI systems may perform well in food detection and categorization, accurate estimation of total energy and nutrient intake remains highly context-dependent and vulnerable to cumulative error across several linked steps, including food identification, ingredient inference, portion estimation, and database matching [6,7,13,14]. However, AI systems were closest to the standard for variety and processing, both of which are comparatively pattern-based and descriptive dimensions of menu evaluation. In contrast, AI produced the largest MAE_kcal, suggesting that coherent textual output and plausible qualitative judgment do not necessarily translate into numerical precision for daily energy intake. This is especially important in pediatrics, where caloric mismatch may have direct implications for growth, adequacy, and long-term metabolic risk, even when the overall dietary description appears convincing [1,11,14].

### 4.1. Interpretation of AI Strengths

Recent comparative studies on AI-generated diet plans support similar findings. A 2026 study in adolescents found substantial underestimation of total energy intake in AI-generated meal plans compared with dietitian-generated plans, together with clinically relevant macronutrient imbalances [15]. Likewise, a 2025 comparison of ChatGPT (OpenAI, version GPT-4)-generated and dietitian-generated clinical diets for obesity and chronic disease reported that AI outputs frequently failed to meet individualized nutritional requirements and occasionally included contraindicated foods [16]. Taken together with the present results, these data suggest that current AI systems may produce outputs that are fluent and persuasive, yet still insufficiently precise for individualized pediatric nutritional decision-making [15,16,17,18].

In qualitative domains, AI showed smaller deviations than at least some of the human groups, particularly for variety and processing. This nuance is important because it aligns with a broader body of evidence suggesting that AI performs especially well in repetitive, pattern-recognition-based, and structurally framed tasks. However, it remains less dependable when contextual synthesis, precise quantification, and individualized clinical interpretation are required [7,13,14]. In the context of nutrition communication, related work has also shown that AI-generated nutrition responses may be perceived as highly coherent, empathetic, and useful, even when expert oversight remains necessary to ensure accuracy and safety [13,19,20].

### 4.2. Human Evaluator Findings

An apparently counterintuitive result was that nutrition specialists showed greater deviation than some physician groups for certain nutritional-quality and standardized ratings. Yet, this should be interpreted carefully. In this study, agreement was defined as proximity to a specific expert-defined reference standard rather than as an absolute measure of professional competence. It is plausible that dietitians and nutrition specialists applied stricter or more nuanced criteria to nutritional quality, especially in borderline cases, leading to divergence from the consensus standard without necessarily indicating inferior clinical reasoning. Therefore, a higher deviation may sometimes reflect a more conservative or more critical evaluative framework [21]. Another important finding was that evaluators who explicitly calculated total kcal tended to show lower deviations in several qualitative and global dimensions and higher binary accuracy. One plausible explanation is that performing a caloric calculation reflects a more systematic analytic approach to menu interpretation, which may improve internal consistency across multiple domains of evaluation. This idea is compatible with the literature suggesting that structured dietary-assessment workflows, including AI-assisted systems, may reduce omissions and improve standardization compared with less explicit or more intuitive assessment strategies [14,18,19]. However, the present results also show that simply providing a kcal estimate is not equivalent to being accurate, because energy-estimation error varied across both human and AI evaluations.

### 4.3. Correlations and Models

The exploratory correlation analyses provide additional context regarding co-occurrence of agreement patterns across domains. Among assessments with an available total-energy estimate, higher MAE kcal was associated with deviations from the expert reference standard for processing, color, and global evaluation ratings. These findings indicate that larger energy-estimation error tended to co-occur with greater deviation in selected other domains. However, the associations are cross-sectional, exploratory, and do not establish directionality or causation [13,14,18].

The multivariable analyses reinforce the importance of evaluator background. Even after adjustment for years of experience, practice setting, domicile region, level of nutritional assessment, level of recommendation, and collaboration with a registered dietitian, evaluator category remained independently associated with multiple outcomes. These exploratory findings suggest that evaluator background may be associated with agreement patterns beyond the measured characteristics included in the models. In practical terms, dietitians, pediatric-focused physicians, physicians with other specialties, and AI systems appear to process the same menu information through partly distinct decision frameworks [21].

### 4.4. Clinical Implications

These findings support a hybrid model rather than a replacement model for AI in pediatric menu assessment. Under structured conditions, AI appears potentially useful for first-pass screening, identification of obvious issues related to variety or processing, and generation of structured preliminary feedback. However, tasks that require individualized recommendations require human supervision, particularly by professionals with formal nutrition training, but, because we did not evaluate clinical workflow integration, more prospective and detailed data are needed. This observation is in line with current reviews of AI in nutrition and dietetics, which consistently describe AI as a promising support tool but emphasize unresolved issues related to validation, transparency, implementation, and safety in real clinical environments [13,14,15,22].

### 4.5. Strengths, Limitations and Future Directions

The strength of the design is that we directly compared multiple categories of human evaluators with a relatively broad range of AI platforms using the same three pediatric cases, the same structured information, and the same standard reference framework. This approach allowed the analysis to go beyond kcal estimation alone and to examine agreement across portions, quantities, binary adequacy indicators, ordinal qualitative dimensions, and standardized scoring systems. At the same time, this strength is also linked to an important methodological limitation: the structured nature of the material likely reduced ambiguity and may have favored reproducibility compared with real-world clinical settings, where dietary information is often incomplete, imprecise, or recall-based.

Several additional limitations should also be acknowledged. First, the study included only healthy children and should not be extrapolated directly to pediatric patients with obesity, malnutrition, food allergy, diabetes, gastrointestinal disease, or other acute and chronic disorders, where nutritional assessment is more individualized and clinically consequential [16]. Second, only three pediatric cases were used, even though they were deliberately chosen to represent clearly different but plausible healthy profiles. The cases were used as fixed standardized assessment vignettes rather than as a random sample of pediatric patients. Multiple evaluators assessed each vignette; therefore, the 344 observations represent evaluator-level assessments and should not be interpreted as 344 independent children. Although each human evaluator completed only one randomly assigned case and all outcomes were calculated relative to a case-specific expert standard, the limited number of vignettes means that case-related heterogeneity could not be fully characterized. The findings should consequently be interpreted as comparative performance across these three structured pediatric scenarios, rather than as population-level estimates applicable to all children, menus, or clinical contexts. Third, the number of evaluators providing total kcal estimates was relatively small. In addition, the reference standard was established through direct discussion and unanimous consensus within an independent multidisciplinary panel comprising three clinical dietitians, two pediatricians, and one school medicine physician. Although the panel reviewed standardized case materials and used nutritional calculations based on the USDA FoodData Central database, independent pre-consensus ratings and formal inter-rater agreement statistics were not obtained. Furthermore, some reference domains, particularly variety, processing level, nutritional quality, color, and attractiveness, necessarily involved professional interpretation. These ratings should therefore be viewed as consensus-based expert reference standards rather than definitive biological ground-truth measures. Fourth, the voluntary recruitment strategy for human evaluators may have introduced self-selection bias. Recruitment through institutional mailing lists, universities, research centers, professional associations, and targeted e-mails may have preferentially reached individuals who were more interested in nutrition, more engaged in continuing professional development, or more confident in performing dietary evaluations. Although recruitment was conducted without prescreening for performance and aimed to include a broad range of professionals involved in pediatric nutrition-related practice, the human evaluator sample may not fully represent the knowledge, skills, or routine assessment practices of all professionals in the target categories. In addition, although participants originated from several Romanian geographic regions, the findings may partly reflect the level and content of nutrition-related training available to the professional groups represented in this national context. Accordingly, the comparative performance of the human evaluator groups should not be assumed to generalize directly to professionals trained or practicing in other countries or healthcare systems, where curricula, scopes of practice, access to dietitians, and routine approaches to pediatric nutrition assessment may differ.

Also, the AI sample was selected pragmatically to reflect the platforms most commonly encountered by parents and healthcare professionals in Romania at the time of the study, rather than representing an exhaustive or globally representative set of systems. Finally, although post hoc corrections were applied where appropriate, the large number of secondary analyses means that isolated significant findings should be interpreted cautiously. AI assessments were not duplicate responses to the same case: each platform/configuration unit generated two assessments for two different pediatric case vignettes. Nevertheless, some configuration units originated from the same AI provider and may have shared underlying model families, infrastructure, or default behavior. The study was designed to capture the range of access modes and configurations realistically available to users, not to estimate within-platform test–retest reliability or isolate the independent effects of provider, model architecture, version, access type, browsing capability, or reasoning mode. Therefore, residual dependence among related configurations and heterogeneity across AI systems cannot be excluded.

Future investigations should include a larger and more diverse set of pediatric cases, with balanced evaluator-by-case allocation and repeated assessment designs that would permit formal modelling of both evaluator-level and case-level sources of variation. Future studies should also expand this framework to pathological pediatric populations, where menu evaluation is more clinically complex and where the consequences of error may be greater. Research should also move beyond agreement with a reference standard and evaluate reproducibility, safety, workflow integration, and clinical usefulness in practice. The key question is not only whether AI can approximate expert judgment under structured conditions, but whether it can improve the consistency and efficiency of pediatric nutrition assessment without compromising individualization, safety, or accountability. Comparative studies embedded in real clinical workflows will be especially important for defining evidence-based roles for AI in pediatric nutrition services.

## 5. Conclusions

This study compared the performance of multiple AI systems and three groups of human professionals in evaluating three standardized pediatric menu case vignettes against case-specific expert-defined reference ratings across quantitative and qualitative domains. Under these highly structured pediatric assessment scenarios, AI systems showed strong agreement with the reference standard for several descriptive and qualitative dimensions, particularly menu variety and processing level, but remained less dependable for precise energy estimation and other quantitatively sensitive tasks.

These findings are limited to the three standardized case vignettes used in this study and should not be generalized directly to the broader pediatric population, to all dietary records, or to clinical decision-making without validation in larger and more heterogeneous pediatric case samples. Overall, these results identify domain-specific patterns of agreement with expert reference standard but do not establish an operational clinical role for AI in pediatric nutrition assessment. They support further research on AI-assisted structured menu review, while emphasizing the need for external validation, reproducibility testing, clinical-safety assessment, and evaluation in diverse real-world pediatric settings before implementation in clinical care.

## Figures and Tables

**Table 1:** Deviations for qualitative ordinal outcomes across evaluator groups.

Outcome	AI (*n* = 84)	Nutrition Specialists (*n* = 108)	Pediatric-Focused Physicians (*n* = 56)	Physicians with Other Specialties (*n* = 88)	Kruskal-Wallis Statistic	*p*-Value
Diff_variety mean ± SD	0.37 ± 0.49	1.15 ± 0.76	1.29 ± 0.89	1.36 ± 0.89	71.45	<0.0001
Median (IQR)	0 (0–1)	1 (1–2)	2 (0–2)	2 (0–2)		
Diff_processing mean ± SD	0.33 ± 0.47	1.22 ± 0.69	1.57 ± 0.74	1.36 ± 0.78	105.5	<0.0001
Median (IQR)	0 (0–1)	1 (1–2)	2 (1–2)	2 (1–2)		
Diff_quality mean ± SD	0.33 ± 0.47	1.00 ± 1.02	0.29 ± 0.46	0.27 ± 0.45	46.68	<0.0001
Median (IQR)	0 (0–1)	1 (0–1)	0 (0–1)	0 (0–1)		
Diff_color mean ± SD	0.43 ± 0.52	0.56 ± 0.74	0.57 ± 0.74	0.45 ± 0.79	2.93	0.4032
Median (IQR)	0 (0–1)	0 (0–1)	0 (0–1)	0 (0–1)		
Diff_attractiveness mean ± SD	0.65 ± 0.48	1.69 ± 0.46	1.43 ± 0.74	1.64 ± 0.78	116.70	<0.0001
Median (IQR)	1 (0–1)	2 (1–2)	2 (1–2)	2 (1–2)		

**Table 2:** Deviations for standardized 1–10 ratings across evaluator groups.

Outcome Mean ± SD; Median	AI (*n* = 84)	Nutrition Specialists (*n* = 116)	Pediatric-Focused Physicians (*n* = 56)	Physicians with Other Specialties (*n* = 88)	Kruskal-Wallis Statistic	*p*-Value
Diff_qualitative	0.90 ± 0.75; 1	2.07 ± 2.03; 1	0.29 ± 0.46; 0	2.00 ± 2.64; 1	66.22	<0.0001
Diff_nutritional	1.56 ± 1.36; 1	1.86 ± 1.26; 2	1.29 ± 2.00; 1	1.36 ± 2.32; 0	34.25	<0.0001
Diff_attractiveness	1.05 ± 0.74; 1	1.69 ± 1.99; 1	0.57 ± 0.74; 0	0.73 ± 0.87; 0	17.88	0.0005

**Table 3:** Exploratory Spearman’s rank correlations between energy-estimation error, evaluator characteristics, and agreement-related deviation outcomes.

Pair	r	95% CI	*p*-Value	Pair	r	95% CI	*p*-Value
MAE_kcal vs. Diff_processing (*n* = 77)	0.402	0.205 to 0.567	<0.0001	MAE_kcal vs. Acc_bin (*n* = 77)	0.003	−0.211 to 0.217	0.976
MAE_kcal vs. Diff_variety (*n* = 77)	0.559	0.391 to 0.690	<0.0001	Experience vs. Diff_variety (*n* = 252)	−0.302	−0.386 to −0.214	<0.0001
MAE_kcal vs. Diff_global (*n* = 77)	−0.499	−0.644 to −0.319	<0.0001	Experience vs. Diff_attractiveness (*n* = 252)	−0.253	−0.338 to −0.163	<0.0001
MAE_kcal vs. Diff_color (*n* = 77)	−0.365	−0.537 to −0.163	0.0004	Dietitian collaboration vs. Diff_attractiveness (*n* = 252)	0.125	0.032 to 0.216	0.0071

**Table 4:** Summary of exploratory multivariable linear regression models for evaluator-agreement outcomes among human evaluators.

Dependent Variable	F (13, 238)	*p*-Value	R^2^	Dependent Variable	F (13, 238)	*p*-Value	R^2^
Diff_portions	10.08	<0.0001	0.3551	Diff_global rating	97.84	<0.0001	0.8424
Diff_quantities	11.30	<0.0001	0.3817	Diff_quantitative (1–10)	15.67	<0.0001	0.4611
Diff_variety	32.31	<0.0001	0.6383	Diff_qualitative (1–10)	30.52	<0.0001	0.6251
Diff_processing	50.40	<0.0001	0.7335	Diff_nutritional (1–10)	101.4	<0.0001	0.8470
Diff_quality	80.21	<0.0001	0.8142	Diff_attractiveness (1–10)	32.02	<0.0001	0.6362
Diff_color	40.72	<0.0001	0.6898	Diff_color (1–10)	73.72	<0.0001	0.8011
Diff_attractiveness	21.60	<0.0001	0.5413	Acc_bin	15.58	<0.0001	0.4598

**Table 5:** Selected exploratory multivariable associations with portion- and quantity-rating deviation among human evaluators.

Predictor	Diff_Portions β (95% CI)	*p*-Value	Diff_Quantities β (95% CI)	*p*-Value
Physicians with other specialties	0.349 (0.194 to 0.503)	<0.0001	0.256 (0.107 to 0.405)	0.0008
Pediatric-focused physician	0.432 (0.226 to 0.639)	<0.0001	0.446 (0.247 to 0.645)	<0.0001
Experience (years)	−0.019 (−0.041 to 0.002)	0.081	−0.026 (−0.047 to −0.005)	0.017

## Data Availability

The anonymized evaluator-level dataset supporting the findings of this study is available from the corresponding author upon reasonable request, subject to institutional ethics approval and applicable data-protection requirements.

## Supplementary Materials

The following supporting information can be downloaded at: https://www.mdpi.com/article/10.3390/nu18183106/s1. Supplementary File S1. Standardized Romanian-language AI prompt and English translation. Supplementary Table S1. Distribution of evaluator assessments by pediatric case vignette and evaluator group. Supplementary Table S2. Number of available evaluator-level assessments for each prespecified outcome, according to evaluator group. Supplementary Table S3. Full exploratory multivariable linear regression results and multicollinearity diagnostics for evaluator-agreement outcomes among human evaluators. Supplementary Table S3A. Exploratory multivariable linear regression model for portion-rating deviation. Supplementary Table S3B. Exploratory multivariable linear regression model for quantity-rating deviation. Supplementary Table S3C. Exploratory multivariable linear regression model for variety-rating deviation. Supplementary Table S3D. Exploratory multivariable linear regression model for processing-level deviation. Supplementary Table S3E. Exploratory multivariable linear regression model for nutritional-quality deviation. Supplementary Table S3F. Exploratory multivariable linear regression model for color-rating deviation. Supplementary Table S3G. Exploratory multivariable linear regression model for attractiveness-rating deviation. Supplementary Table S3H. Exploratory multivariable linear regression model for global-class deviation. Supplementary Table S3I. Exploratory multivariable linear regression model for standardized quantitative-rating deviation. Supplementary Table S3J. Exploratory multivariable linear regression model for standardized qualitative-rating deviation. Supplementary Table S3K. Exploratory multivariable linear regression model for standardized nutritional-rating deviation. Supplementary Table S3L. Exploratory multivariable linear regression model for standardized attractiveness-rating deviation. Supplementary Table S3M. Exploratory multivariable linear regression model for standardized coloristic-rating deviation. Supplementary Table S3N. Exploratory multivariable linear regression model for binary agreement score (Acc_bin). Supplementary Table S4. AI platforms with model/version, configuration and allocated case.

## Abbreviations

The following abbreviations are used in this manuscript:

Acc_bin	Binary agreement with reference indicators
AI	Artificial intelligence
BMI	Body Mass Index
CI	Confidence interval
LLM	Large Language Model
MAE_kcal	The absolute difference between the evaluator’s estimated total daily energy intake and the standard for the corresponding case
*p* value	Probability value
q value	*p*-value adjusted for the False Discovery Rate (FDR)
USDA	United States of America Department of Agriculture
