# Supplementary material for: Domain-Dependent Performance of Human Experts and AI Systems in Pediatric Menu Evaluation
Source: Nutrients. 2026 Sep 21;18(18):3106. doi: 10.3390/nu18183106 (PMC13610824; doi:10.3390/nu18183106)
Supplement: Supplementary file 1 [file nutrients-18-03106-s001.zip › nutrients-4538103-supplementary.pdf]

nutrients

MDPI

Supplementary File S1. Standardized Romanian-language AI prompt and English translation

Ro: Analizează jurnalul alimentar atașat din punct de vedere nutrițional în funcție de informațiile pe care le deții. Nu ai impus un model unic de calcul, poți utiliza instrumentele proprii de analiză, dar respectă exact structura chestionarului de mai jos:

1. Evaluare cantitativă – estimarea aportului alimentar (porții, cantități, echilibru nutrițional):

2. Evaluare calitativă – aprecierea calității alimentelor și a modului de prezentare (varietate, procesare, coloristică, atractivitate):

3. Evaluare globală pe clase (alege răspunsul potrivit, fără alte detalii):
- Clasa 1: Corespunzător și nutritiv, general (meniul este echilibrat și potrivit pentru majoritatea copiilor de vârstă similară)
- Clasa 2: Corespunzător în general, dar nepotrivit cazului dat (ar fi un meniu bun în alte contexte, dar nu se potrivește particularităților copilului analizat)
- Clasa 3: Corespunzător cazului dat, dar necorespunzător în general (adaptat unui caz special, dar nu ar fi un model sănătos pentru un copil tipic)
- Clasa 4: Inadecvat (meniul prezintă dezechilibre majore, indiferent de contextul copilului)
- Clasa 5: Nu sunt în măsură să evaluez acest meniu/caz

4. Evaluare standardizată (oferă nota potrivită, unde 1 = slab, 10 = excelent, fără alte detalii):
- Cantitativ
- Calitativ
- Nutritiv
- Atractiv
- Coloristic

5. În ce măsură sunteți implicat/ă în evaluarea nutrițională? (alege răspunsurile potrivite, fără alte detalii):
- Nu sunt implicat
- Recomand consult dietetic
- Fac o evaluare dietetică de bază
- Fac o evaluare dietetică complexă
- Fac recomandări dietetice de bază
- Fac recomandări dietetice complexe

 https://doi.org/10.3390/nu18183106

En: Analyze the food diary shown from a nutritional perspective, based on the information available to you. No single calculation model has been imposed; you may use your own analytical tools, but follow exactly the questionnaire structure below:

1. Quantitative evaluation – estimation of dietary intake (portion sizes, quantities, nutritional balance):

2. Qualitative evaluation – appraisal of food quality and presentation (variety, processing level, color profile, attractiveness):

3. Overall class-based evaluation (select the appropriate answer, without additional details):

- Class 1: Appropriate and nutritious in general (the menu is balanced and suitable for most children of a similar age)
- Class 2: Generally appropriate, but inappropriate for the specific case (it would be a good menu in other contexts but does not fit the characteristics of the child being evaluated)
- Class 3: Appropriate for the specific case, but generally inappropriate (adapted to a special case but would not be a healthy model for a typical child)
- Class 4: Inappropriate (the menu presents major imbalances regardless of context)
- Class 5: I am not able to evaluate this menu/case

4. Standardized evaluation (provide the appropriate score, where 1 = poor and 10 = excellent, without additional details):

- Quantitative
- Qualitative
- Nutritional
- Attractiveness
- Color profile

5. To what extent are you involved in nutritional assessment? (select the appropriate responses, without additional details):

- I am not involved
- I recommend consultation with a dietitian
- I perform a basic dietary assessment
- I perform a comprehensive dietary assessment
- I provide basic dietary recommendations
- I provide comprehensive dietary recommendations

Note: The prompt was presented in Romanian to all AI platform/configuration units. The pediatric case-specific clinical and anthropometric information, food diary, food weights and portion descriptions, meal photographs, and meal-serving information were provided together with this standardized prompt. No external nutritional database was manually supplied by the investigators. Platform-specific default features, including availability of web browsing or reasoning modes, were retained as available within the selected configuration.

https://doi.org/10.3390/nu18183106

Supplementary Table S1. Distribution of evaluator assessments by pediatric case vignette and evaluator group

Evaluator group	*Case 1*	*Case 2*	*Case 3*	*Total*
AI systems	28	28	28	84
Nutrition specialists	44	28	44	116
Pediatric-focused physicians	16	16	24	56
Physicians with other specialties	24	8	56	88
Total	**112**	**80**	**152**	**344**

Each human evaluator completed one assessment for one randomly assigned pediatric case vignette. AI evaluations were conducted using the same standardized case materials. The table describes the distribution of evaluator-level assessments and does not represent the number of pediatric participants.

Supplementary Table S2. Number of available evaluator-level assessments for each prespecified outcome, according to evaluator group

Outcome	***AI systems***	***Nutrition specialists***	***Pediatric-focused physicians***	***Physicians with other specialties***	***Total***
All included assessments	84	116	56	88	344
MAE_kcal	61	8	0	8	77
Portion deviation	84	116	56	88	344
Quantity deviation	84	116	56	88	344
Variety deviation	84	108	56	88	336
Processing deviation	84	108	56	88	336
Nutritional-quality deviation	84	108	56	88	336
Color deviation	84	108	56	88	336
Attractiveness deviation	84	116	56	88	344
Global-class deviation	84	116	56	88	344
Standardized quantitative-rating deviation	84	116	56	88	344
Standardized qualitative-rating deviation	84	116	56	88	344
Standardized nutritional-rating deviation	84	116	56	88	344
Standardized attractiveness-rating deviation	84	116	56	88	344
Standardized coloristic-rating deviation	84	116	56	88	344
Binary agreement score (Acc_bin)	84	116	56	88	344

Supplementary Table S3. Full exploratory multivariable linear regression results and multicollinearity diagnostics for evaluator-agreement outcomes among human evaluators

All models were fitted among human evaluators (n = 252) using ordinary least-squares multiple linear regression in GraphPad Prism 9. The reference categories were nutrition specialists for professional category, Central for geographic region, and public for practice setting. Professional category was represented by indicator variables for pediatric-focused physicians and physicians with other specialties. Geographic regions shown are compared with the Central region. Practice settings shown are compared with public practice. Positive regression coefficients indicate greater deviation from the expert reference standard, except for binary agreement (Acc_bin), for which positive coefficients indicate higher agreement. Models included professional category, years of professional experience, geographic region, practice setting, level of nutritional-assessment involvement, level of dietary-recommendation involvement, and collaboration with a registered dietitian. Because several outcomes were derived from bounded or ordinal scales, the regression analyses were exploratory and should not be interpreted as causal. VIF values ranged from 1.29 to 3.88, indicating no severe multicollinearity.

Abbreviations: B, unstandardized regression coefficient; SE, standard error; CI, confidence interval; VIF, variance inflation factor; Accbin, binary agreement with case-specific reference indicators; RD, registered dietitian.

Supplementary Table S3A. Exploratory multivariable linear regression model for portion-rating deviation

Predictor	*B*	*SE*	*95% CI*	*p value*	*VIF*
Intercept	−0.5610	0.1792	−0.9140 to −0.2080	0.0020	—
Physicians with other specialties	0.3488	0.0785	0.1942 to 0.5033	<0.0001	1.749
Pediatric-focused physicians	0.4323	0.1049	0.2257 to 0.6389	<0.0001	2.375
Professional experience, years	−0.0195	0.0111	−0.0414 to 0.0024	0.0813	1.534
Southeast region	0.1356	0.1159	−0.0927 to 0.3638	0.2431	1.446
Northwest region	−0.7151	0.1161	−0.9437 to −0.4865	<0.0001	1.866
North region	0.2624	0.1272	0.0119 to 0.5129	0.0402	2.241
South region	−1.0170	0.1597	−1.3320 to −0.7024	<0.0001	1.895
Southwest region	0.0876	0.1319	−0.1723 to 0.3476	0.5072	1.294
Mixed public/private practice	−0.6664	0.1525	−0.9669 to −0.3659	<0.0001	3.884
Private practice	0.1590	0.0984	−0.0348 to 0.3528	0.1073	1.615
Nutritional-assessment involvement level	0.2201	0.0359	0.1494 to 0.2909	<0.0001	2.099
Dietary-recommendation involvement level	0.2561	0.0477	0.1622 to 0.3499	<0.0001	3.206
Collaboration with registered dietitian	0.0323	0.0117	0.0092 to 0.0555	0.0063	1.526
Model statistics: $F(13,238)=10.08$; $p < 0.0001$; $R^2=0.3551$					

https://doi.org/10.3390/nu18183106

Supplementary Table S3B. Exploratory multivariable linear regression model for quantity-rating deviation.

Predictor	*B*	*SE*	*95% CI*	*p value*	*VIF*
Intercept	−0.5748	0.1726	−0.9148 to −0.2349	0.0010	—
Physicians with other specialties	0.2560	0.0756	0.1071 to 0.4049	0.0008	1.749
Pediatric-focused physicians	0.4462	0.1010	0.2473 to 0.6452	<0.0001	2.375
Professional experience, years	−0.0258	0.0107	−0.0469 to −0.0047	0.0168	1.534
Southeast region	0.1785	0.1116	−0.0413 to 0.3983	0.1110	1.446
Northwest region	−0.7022	0.1118	−0.9224 to −0.4820	<0.0001	1.866
North region	0.2610	0.1225	0.0197 to 0.5023	0.0342	2.241
South region	−1.0790	0.1538	−1.3820 to −0.7757	<0.0001	1.895
Southwest region	0.1178	0.1271	−0.1325 to 0.3682	0.3547	1.294
Mixed public/private practice	−0.7363	0.1469	−1.0260 to −0.4469	<0.0001	3.884
Private practice	0.1502	0.0948	−0.0365 to 0.3368	0.1144	1.615
Nutritional-assessment involvement level	0.2576	0.0346	0.1895 to 0.3258	<0.0001	2.099
Dietary-recommendation involvement level	0.2536	0.0459	0.1632 to 0.3440	<0.0001	3.206
Collaboration with registered dietitian	0.0301	0.0113	0.0078 to 0.0524	0.0082	1.526
Model statistics: F(13,238)=11.30; p < 0.0001; R^2=0.3817					

Supplementary Table S3C. Exploratory multivariable linear regression model for variety-rating deviation

Predictor	*B*	*SE*	*95% CI*	*p value*	*VIF*
Intercept	−0.0057	0.2062	−0.4120 to 0.4005	0.9779	—
Physicians with other specialties	0.0163	0.0903	−0.1617 to 0.1941	0.8574	1.749
Pediatric-focused physicians	−0.0966	0.1207	−0.3344 to 0.1411	0.4242	2.375
Professional experience, years	0.1379	0.0128	0.1127 to 0.1631	<0.0001	1.534
Southeast region	−0.1698	0.1334	−0.4325 to 0.0929	0.2041	1.446
Northwest region	0.5576	0.1336	0.2945 to 0.8208	<0.0001	1.866
North region	0.6787	0.1464	0.3903 to 0.9670	<0.0001	2.241
South region	−0.9113	0.1838	−1.2730 to −0.5492	<0.0001	1.895
Southwest region	−1.0140	0.1519	−1.3130 to −0.7144	<0.0001	1.294
Mixed public/private practice	−1.6990	0.1756	−2.0440 to −1.3530	<0.0001	3.884
Private practice	0.4003	0.1132	0.1772 to 0.6234	0.0005	1.615
Nutritional-assessment involvement level	0.1149	0.0413	0.0334 to 0.1963	0.0059	2.099
Dietary-recommendation involvement level	0.2584	0.0548	0.1504 to 0.3665	<0.0001	3.206
Collaboration with registered dietitian	−0.0144	0.0135	−0.0410 to 0.0122	0.2883	1.526
Model statistics: F(13,238)=32.3; p < 0.0001; R^2=0.6383					

Supplementary Table S3D. Exploratory multivariable linear regression model for processing-level deviation

Predictor	*B*	*SE*	*95% CI*	*p value*	*VIF*
Intercept	0.4255	0.1564	0.1174 to 0.7337	0.0070	—
Physicians with other specialties	−0.1181	0.0685	−0.2531 to 0.0168	0.0859	1.749
Pediatric-focused physicians	0.2318	0.0916	0.0514 to 0.4121	0.0120	2.375
Professional experience, years	0.1833	0.0097	0.1641 to 0.2024	<0.0001	1.534
Southeast region	−0.3031	0.1012	−0.5024 to −0.1039	0.0030	1.446
Northwest region	0.4542	0.1013	0.2546 to 0.6539	<0.0001	1.866
North region	−0.2458	0.1110	−0.4645 to −0.0271	0.0278	2.241
South region	1.3830	0.1394	1.1080 to 1.6570	<0.0001	1.895
Southwest region	−1.2070	0.1152	−1.4330 to −0.9796	<0.0001	1.294
Mixed public/private practice	1.0090	0.1332	0.7465 to 1.2710	<0.0001	3.884
Private practice	0.1944	0.0859	0.0252 to 0.3636	0.0245	1.615
Nutritional-assessment involvement level	−0.2279	0.0314	−0.2897 to −0.1661	<0.0001	2.099
Dietary-recommendation involvement level	−0.3173	0.0416	−0.3992 to −0.2353	<0.0001	3.206
Collaboration with registered dietitian	0.0283	0.0102	0.0081 to 0.0485	0.0062	1.526
Model statistics: $F(13,238)=50.40$, $p < 0.0001$; $R^2=0.7335$					

Supplementary Table S3E. Exploratory multivariable linear regression model for nutritional-quality deviation

Predictor	*B*	*SE*	*95% CI*	*p value*	*VIF*
Intercept	1.5020	0.1467	1.2130 to 1.7910	<0.0001	—
Physicians with other specialties	−0.2461	0.0642	−0.3727 to −0.1196	0.0002	1.749
Pediatric-focused physicians	−0.7821	0.0859	−0.9512 to −0.6129	<0.0001	2.375
Professional experience, years	−0.1506	0.0091	−0.1686 to −0.1327	<0.0001	1.534
Southeast region	0.4006	0.0949	0.2137 to 0.5874	<0.0001	1.446
Northwest region	0.0185	0.0950	−0.1687 to 0.2057	0.8458	1.866
North region	0.9527	0.1041	0.7476 to 1.1580	<0.0001	2.241
South region	−1.0660	0.1307	−1.3230 to −0.8080	<0.0001	1.895
Southwest region	0.4105	0.1080	0.1977 to 0.6233	0.0002	1.294
Mixed public/private practice	−0.8394	0.1249	−1.0850 to −0.5934	<0.0001	3.884
Private practice	−0.1653	0.0805	−0.3239 to −0.0066	0.0413	1.615
Nutritional-assessment involvement level	0.1837	0.0294	0.1257 to 0.2416	<0.0001	2.099
Dietary-recommendation involvement level	0.3694	0.0390	0.2926 to 0.4462	<0.0001	3.206
Collaboration with registered dietitian	−0.0409	0.0096	−0.0598 to −0.0220	<0.0001	1.526
Model statistics: $F(13,238)=80.21$, $p < 0.0001$; $R^2=0.8142$					

Supplementary Table S3F. Exploratory multivariable linear regression model for color-rating deviation

Predictor	*B*	*SE*	*95% CI*	*p value*	*VIF*
Intercept	1.8320	0.1722	1.4930 to 2.1720	<0.0001	—
Physicians with other specialties	−0.1405	0.0754	−0.2891 to 0.0080	0.0635	1.749
Pediatric-focused physicians	0.4187	0.1008	0.2202 to 0.6172	<0.0001	2.375
Professional experience, years	−0.1684	0.0107	−0.1894 to −0.1473	<0.0001	1.534
Southeast region	0.4132	0.1113	0.1939 to 0.6325	0.0003	1.446
Northwest region	−0.5617	0.1115	−0.7814 to −0.3420	<0.0001	1.866
North region	0.2304	0.1222	−0.0104 to 0.4711	0.0606	2.241
South region	−0.3985	0.1535	−0.7008 to −0.0962	0.0100	1.895
Southwest region	1.1600	0.1268	0.9100 to 1.4090	<0.0001	1.294
Mixed public/private practice	−0.0931	0.1466	−0.3818 to 0.1957	0.5261	3.884
Private practice	−1.0290	0.0945	−1.2150 to −0.8427	<0.0001	1.615
Nutritional-assessment involvement level	0.0391	0.0345	−0.0289 to 0.1071	0.2581	2.099
Dietary-recommendation involvement level	−0.0950	0.0458	−0.1852 to −0.0048	0.0390	3.206
Collaboration with registered dietitian	0.0060	0.0113	−0.0162 to 0.0282	0.5936	1.526
Model statistics: $F(13,238)=40.72$; $p < 0.0001$; $R^2=0.6898$					

Supplementary Table S3G. Exploratory multivariable linear regression model for attractiveness-rating deviation

Predictor	*B*	*SE*	*95% CI*	*p value*	*VIF*
Intercept	1.8820	0.1807	1.5260 to 2.2380	<0.0001	—
Physicians with other specialties	−0.1122	0.0791	−0.2681 to 0.0437	0.1577	1.749
Pediatric-focused physicians	−0.9045	0.1058	−1.1130 to −0.6961	<0.0001	2.375
Professional experience, years	0.0525	0.0112	0.0304 to 0.0746	<0.0001	1.534
Southeast region	−0.3197	0.1169	−0.5499 to −0.0895	0.0067	1.446
Northwest region	0.7406	0.1171	0.5100 to 0.9712	<0.0001	1.866
North region	−0.6193	0.1283	−0.8720 to −0.3666	<0.0001	2.241
South region	−0.6334	0.1611	−0.9507 to −0.3161	0.0001	1.895
Southwest region	−0.5341	0.1331	−0.7962 to −0.2719	<0.0001	1.294
Mixed public/private practice	0.2717	0.1539	−0.0314 to 0.5748	0.0787	3.884
Private practice	0.5906	0.0992	0.3951 to 0.7861	<0.0001	1.615
Nutritional-assessment involvement level	−0.1111	0.0362	−0.1824 to −0.0397	0.0024	2.099
Dietary-recommendation involvement level	0.1547	0.0481	0.0600 to 0.2494	0.0015	3.206
Collaboration with registered dietitian	−0.0457	0.0118	−0.0690 to −0.0224	0.0001	1.526
Model statistics: $F(13,238)=21.60$; $p < 0.0001$; $R^2=0.5413$					

Supplementary Table S3H. Exploratory multivariable linear regression model for global-class deviation

Predictor	*B*	*SE*	*95% CI*	*p value*	*VIF*
Intercept	4.7890	0.5140	3.7760 to 5.8010	<0.0001	—
Physicians with other specialties	1.8140	0.2251	1.3710 to 2.2580	<0.0001	1.749
Pediatric-focused physicians	2.1410	0.3008	1.5480 to 2.7330	<0.0001	2.375
Professional experience, years	0.5531	0.0319	0.4903 to 0.6159	<0.0001	1.534
Southeast region	1.1070	0.3324	0.4519 to 1.7610	0.0010	1.446
Northwest region	0.9242	0.3329	0.2683 to 1.5800	0.0059	1.866
North region	−1.3910	0.3648	−2.1100 to −0.6722	0.0002	2.241
South region	5.8170	0.4581	4.9150 to 6.7200	<0.0001	1.895
Southwest region	−5.3470	0.3785	−6.0930 to −4.6020	<0.0001	1.294
Mixed public/private practice	5.9220	0.4376	5.0600 to 6.7840	<0.0001	3.884
Private practice	−1.7830	0.2822	−2.3390 to −1.2280	<0.0001	1.615
Nutritional-assessment involvement level	−1.3580	0.1030	−1.5610 to −1.1550	<0.0001	2.099
Dietary-recommendation involvement level	−1.4280	0.1367	−1.6970 to −1.1580	<0.0001	3.206
Collaboration with registered dietitian	−0.0529	0.0337	−0.1192 to 0.0134	0.1171	1.526
Model statistics: F(13,238)=97.84; p < 0.0001; R²=0.8424					

Supplementary Table S3I. Exploratory multivariable linear regression model for standardized quantitative-rating deviation

Predictor	*B*	*SE*	*95% CI*	*p value*	*VIF*
Intercept	2.3160	0.4475	1.4350 to 3.1980	<0.0001	—
Physicians with other specialties	0.5927	0.1960	0.2067 to 0.9788	0.0028	1.749
Pediatric-focused physicians	−0.7568	0.2619	−1.2730 to −0.2408	0.0042	2.375
Professional experience, years	−0.2769	0.0278	−0.3315 to −0.2222	<0.0001	1.534
Southeast region	−0.0606	0.2894	−0.6306 to 0.5095	0.8344	1.446
Northwest region	0.2908	0.2899	−0.2802 to 0.8618	0.3168	1.866
North region	0.9138	0.3176	0.2881 to 1.5400	0.0044	2.241
South region	0.1549	0.3988	−0.6308 to 0.9407	0.6980	1.895
Southwest region	−0.0299	0.3295	−0.6790 to 0.6192	0.9278	1.294
Mixed public/private practice	0.4386	0.3810	−0.3119 to 1.1890	0.2508	3.884
Private practice	0.3033	0.2457	−0.1807 to 0.7873	0.2183	1.615
Nutritional-assessment involvement level	0.0982	0.0897	−0.0786 to 0.2749	0.2749	2.099
Dietary-recommendation involvement level	0.0080	0.1190	−0.2264 to 0.2424	0.9464	3.206
Collaboration with registered dietitian	0.0753	0.0293	0.0176 to 0.1330	0.0108	1.526
Model statistics: F(13,238)=15.67, p < 0.0001; R²=0.4611					

Supplementary Table S3J. Exploratory multivariable linear regression model for standardized qualitative-rating deviation

Predictor	*B*	*SE*	*95% CI*	*p value*	*VIF*
Intercept	3.2480	0.5537	2.1570 to 4.3390	<0.0001	—
Physicians with other specialties	0.3478	0.2425	−0.1299 to 0.8256	0.1528	1.749
Pediatric-focused physicians	−1.1360	0.3241	−1.7740 to −0.4973	0.0005	2.375
Professional experience, years	−0.4558	0.0344	−0.5235 to −0.3881	<0.0001	1.534
Southeast region	0.0243	0.3581	−0.6811 to 0.7297	0.9459	1.446
Northwest region	−1.3920	0.3587	−2.0980 to −0.6849	0.0001	1.866
North region	1.9440	0.3931	1.1690 to 2.7180	<0.0001	2.241
South region	−1.3550	0.4936	−2.3270 to −0.3823	0.0065	1.895
Southwest region	−0.1493	0.4078	−0.9526 to 0.6540	0.7146	1.294
Mixed public/private practice	0.0113	0.4715	−0.9175 to 0.9401	0.9809	3.884
Private practice	−0.7570	0.3040	−1.3560 to −0.1580	0.0135	1.615
Nutritional-assessment involvement level	0.3316	0.1110	0.1129 to 0.5503	0.0031	2.099
Dietary-recommendation involvement level	−0.2405	0.1473	−0.5306 to 0.0496	0.1038	3.206
Collaboration with registered dietitian	0.2063	0.0363	0.1348 to 0.2777	<0.0001	1.526
Model statistics: F(13,238)=30.52; p < 0.0001; R²=0.6251					

Supplementary Table S3K. Exploratory multivariable linear regression model for standardized nutritional-rating deviation

Predictor	*B*	*SE*	*95% CI*	*p value*	*VIF*
Intercept	5.9690	0.3009	5.3770 to 6.5620	<0.0001	—
Physicians with other specialties	−0.6599	0.1318	−0.9195 to −0.4003	<0.0001	1.749
Pediatric-focused physicians	−0.3822	0.1761	−0.7292 to −0.0352	0.0310	2.375
Professional experience, years	−0.5689	0.0187	−0.6057 to −0.5322	<0.0001	1.534
Southeast region	−0.5567	0.1946	−0.9400 to −0.1733	0.0046	1.446
Northwest region	−1.9150	0.1949	−2.2990 to −1.5310	<0.0001	1.866
North region	−0.3112	0.2136	−0.7320 to 0.1096	0.1465	2.241
South region	−4.0880	0.2682	−4.6170 to −3.5600	<0.0001	1.895
Southwest region	1.6350	0.2216	1.1980 to 2.0710	<0.0001	1.294
Mixed public/private practice	−2.3720	0.2562	−2.8760 to −1.8670	<0.0001	3.884
Private practice	−1.1650	0.1652	−1.4910 to −0.8398	<0.0001	1.615
Nutritional-assessment involvement level	0.4511	0.0603	0.3323 to 0.5700	<0.0001	2.099
Dietary-recommendation involvement level	0.3265	0.0800	0.1688 to 0.4841	<0.0001	3.206
Collaboration with registered dietitian	−0.0223	0.0197	−0.0611 to 0.0166	0.2600	1.526
Model statistics: F(13,238)=101.40, p < 0.0001; R²=0.8470					

https://doi.org/10.3390/nu18183106

Supplementary Table S3L. Exploratory multivariable linear regression model for standardized attractiveness-rating deviation

Predictor	*B*	*SE*	*95% CI*	*p value*	*VIF*
Intercept	1.8420	0.3852	1.0830 to 2.6010	<0.0001	—
Physicians with other specialties	−0.4459	0.1687	−0.7782 to −0.1137	0.0087	1.749
Pediatric-focused physicians	−2.1470	0.2254	−2.5910 to −1.7030	<0.0001	2.375
Professional experience, years	−0.2189	0.0239	−0.2660 to −0.1718	<0.0001	1.534
Southeast region	−0.0240	0.2491	−0.5147 to 0.4666	0.9232	1.446
Northwest region	1.0610	0.2495	0.5691 to 1.5520	<0.0001	1.866
North region	0.6815	0.2734	0.1429 to 1.2200	0.0134	2.241
South region	−0.4169	0.3433	−1.0930 to 0.2595	0.2259	1.895
Southwest region	−0.2403	0.2836	−0.7991 to 0.3184	0.3977	1.294
Mixed public/private practice	−1.8730	0.3279	−2.5190 to −1.2270	<0.0001	3.884
Private practice	0.5836	0.2115	0.1670 to 1.0000	0.0062	1.615
Nutritional-assessment involvement level	0.5218	0.0772	0.3696 to 0.6739	<0.0001	2.099
Dietary-recommendation involvement level	0.6289	0.1024	0.4271 to 0.8307	<0.0001	3.206
Collaboration with registered dietitian	−0.0365	0.0252	−0.0861 to 0.0132	0.1496	1.526
Model statistics: $F(13,238)=32.02$; $p < 0.0001$; $R^2=0.6362$					

Supplementary Table S3M. Exploratory multivariable linear regression model for standardized coloristic-rating deviation

Predictor	*B*	*SE*	*95% CI*	*p value*	*VIF*
Intercept	6.3030	0.4143	5.4870 to 7.1190	<0.0001	—
Physicians with other specialties	0.4792	0.1814	0.1218 to 0.8365	0.0088	1.749
Pediatric-focused physicians	0.8577	0.2425	0.3801 to 1.3350	0.0005	2.375
Professional experience, years	0.5783	0.0257	0.5277 to 0.6289	<0.0001	1.534
Southeast region	−0.0187	0.2679	−0.5464 to 0.5091	0.9444	1.446
Northwest region	0.9715	0.2684	0.4429 to 1.5000	0.0004	1.866
North region	1.6640	0.2941	1.0850 to 2.2430	<0.0001	2.241
South region	3.7580	0.3693	3.0300 to 4.4850	<0.0001	1.895
Southwest region	−3.9050	0.3051	−4.5060 to −3.3040	<0.0001	1.294
Mixed public/private practice	3.8560	0.3527	3.1620 to 4.5510	<0.0001	3.884
Private practice	−0.8396	0.2275	−1.2880 to −0.3915	0.0003	1.615
Nutritional-assessment involvement level	−0.8559	0.0831	−1.0200 to −0.6923	<0.0001	2.099
Dietary-recommendation involvement level	−1.1080	0.1102	−1.3250 to −0.8910	<0.0001	3.206
Collaboration with registered dietitian	−0.0563	0.0271	−0.1097 to −0.0029	0.0390	1.526
Model statistics: $F(13,238)=73.72$; $p < 0.0001$; $R^2=0.8011$					

Supplementary Table S3N. Exploratory multivariable linear regression model for binary agreement score (Acc_bin)

Predictor	***B***	***SE***	***95% CI***	***p value***	***VIF***
Intercept	0.3508	0.0781	0.1970 to 0.5045	<0.0001	—
Physicians with other specialties	−0.2419	0.0342	−0.3092 to −0.1746	<0.0001	1.749
Pediatric-focused physicians	−0.1586	0.0457	−0.2486 to −0.0687	0.0006	2.375
Professional experience, years	0.0109	0.0048	0.0014 to 0.0205	0.0251	1.534
Southeast region	−0.0686	0.0505	−0.1680 to 0.0309	0.1757	1.446
Northwest region	0.2001	0.0506	0.1005 to 0.2997	<0.0001	1.866
North region	−0.3589	0.0554	−0.4681 to −0.2498	<0.0001	2.241
South region	−0.0239	0.0696	−0.1609 to 0.1132	0.7317	1.895
Southwest region	−0.1332	0.0575	−0.2465 to −0.0200	0.0213	1.294
Mixed public/private practice	−0.2728	0.0665	−0.4038 to −0.1419	<0.0001	3.884
Private practice	−0.1359	0.0429	−0.2203 to −0.0515	0.0017	1.615
Nutritional-assessment involvement level	0.0407	0.0157	0.0099 to 0.0715	0.0099	2.099
Dietary-recommendation involvement level	0.1000	0.0208	0.0591 to 0.1408	<0.0001	3.206
Collaboration with registered dietitian	−0.0072	0.0051	−0.0173 to 0.0028	0.1583	1.526
Model statistics: $F(13,238)=15.58$, $p < 0.0001$; $R^2=0.4598$					

Supplementary Table S4. AI platforms with model/version, configuration and allocated case

AI platform	*Access tier*	*Model/version*	*Configuration*	*Case*
ChatGPT (OpenAI)	Free	GPT-5.4	Default/automatic mode	1
ChatGPT (OpenAI)	Paid	GPT-5.4	Thinking	2
Claude (Anthropic)	Free	Claude Haiku 4.5	Standard	3
Claude (Anthropic)	Paid	Claude Haiku 4.5	Extended Thinking	1
DeepSeek	Free	DeepSeek V3.2	No Deep Thinking + No Smart Search	2
DeepSeek	Free	DeepSeek V3.2	Deep Thinking + Smart Search	3
Gemini (Google)	Free	Gemini	Default/automatic mode	1
Gemini (Google)	Paid	Gemini Ultra Pro	Pro	2
Grok (xAI)	Paid	Grok 4.1	Rapid	3
Grok (xAI)	Paid	Grok 4.1	Expert – Thinking	1
Perplexity	Pro	Sonar	Standard	2
Perplexity	Pro	Sonar	Deep Research	3
Microsoft Copilot	Free	GPT-based Copilot	Default	1
Microsoft Copilot	Paid	GPT-based Copilot	Advanced/priority access	2
Meta AI	Free	Llama 4 Scout	Standard	3
Meta AI	Paid/Advanced	Llama 4 Maverick	Advanced	1
Mistral Le Chat	Free	Mistral Large	Standard	2
Mistral Le Chat	Paid/Pro	Mistral Large	Pro	3
Qwen	Free	Qwen3	Standard	1
Qwen	Free/Advanced	Qwen3-VL	Vision	2
Kimi	Free	Kimi K2	Standard	3
Kimi	Paid	Kimi K2	Advanced	1
Poe	Free	User-selected multimodal model	Standard access	2
Poe	Paid	User-selected multimodal model	Premium access	3
Jenni AI	Free	Proprietary/third-party LLM	Standard	1
Jenni AI	Paid	Proprietary/third-party LLM	Premium	2
SciSpace	Free	SciSpace Copilot	Standard	3
SciSpace	Premium	SciSpace Copilot	Premium	1
Elicit	Free	Elicit AI	Standard	2
Elicit	Paid	Elicit AI	Advanced	3
QuillBot	Free	QuillBot AI	Standard	1
QuillBot	Premium	QuillBot AI	Premium	2
Foodvisor	Free	Food recognition AI	Standard	3
Foodvisor	Premium	Food recognition AI	Premium	1
MyFitnessPal	Free	Food database / recognition	Standard	2
MyFitnessPal	Premium	Meal Scan	AI image recognition	3

AI platform	*Access tier*	*Model/version*	*Configuration*	*Case*
YAZIO	Free	AI food recognition	Standard	1
YAZIO	Premium	AI food recognition	Premium	2
Lifesum	Free	AI food recognition	Standard	3
Lifesum	Premium	AI food recognition	Premium	1
Lose It!	Free	Snap It	Standard	2
Lose It!	Premium	Snap It	Premium	3
Cal AI	Free/Trial	Proprietary food-image AI	Standard	1
Cal AI	Premium	Proprietary food-image AI	Premium	2
SnapCalorie	Free/Trial	Proprietary food-recognition AI	Standard	3
SnapCalorie	Premium	Proprietary food-recognition AI	Premium	1
Bitesnap	Free	Bitesnap AI	Standard	2
Bitesnap	Premium	Bitesnap AI	Premium	3
Nutrola	Free/Trial	Proprietary food-recognition AI	Standard	1
Nutrola	Premium	Proprietary food-recognition AI	Premium	2
FoodBase	Free/Demo	Food recognition model	Standard	3
FoodBase	Paid/API	Food recognition model	Full API	1
TastyAPI	Free/Demo	Food-analysis AI	Standard	2
TastyAPI	Paid/API	Food-analysis AI	Full API	3
Edamam	Free/Demo	Food Image Analysis	Standard	1
Edamam	Paid/API	Food Image Analysis	Full API	2
Oura	Free	Oura Advisor	Standard AI health analysis	3
Oura	Paid	Oura Advisor	Personalized analysis using Oura health data	1
WHOOP	Free/Included	WHOOP AI Guidance / Coach	Standard health & fitness guidance	2
WHOOP	Paid	WHOOP AI Guidance / Coach	Personalized coaching using Sleep, Strain & Recovery	3
Fitbit / Google Health	Free	Personal Health Coach	Gemini-powered health & fitness guidance	1
Fitbit / Google Health	Premium	Personal Health Coach	Gemini-powered personalized coaching	2
Garmin Connect	Free	Garmin AI / Active Intelligence	Standard health & fitness insights	3
Garmin Connect	Connect+	Active Intelligence	Advanced performance & nutrition insights	1

https://doi.org/10.3390/nu18183106

AI platform	*Access tier*	*Model/version*	*Configuration*	*Case*
Apple Watch / Apple Intelligence	Standard	Apple Intelligence	Health & fitness analysis	2
Apple Watch / Apple Intelligence	Apple Intelligence-enabled	Workout Buddy	Personalized real-time fitness coaching	3
Samsung Health / Galaxy AI	Free	Samsung Health AI	Health/fitness insights	1
Samsung Health / Galaxy AI	Premium/Advanced	Galaxy AI	Personalized health & wellness insights	2
Ultrahuman	Free	Ultrahuman AI	Ring-based health insights	3
Ultrahuman	Paid/Advanced	Ultrahuman AI	Personalized metabolic/recovery insights	1
Withings	Free	Withings Health Insights	Standard health-data interpretation	2
Withings	Withings+	Withings Intelligence	Generative-AI health assistant	3
RingConn	Free	RingConn AI/health insights	Standard health interpretation	1
RingConn	Premium/Advanced	RingConn AI	Advanced health insights	2
Amazfit / Zepp	Free	Zepp AI	Standard health/fitness insights	3
Amazfit / Zepp	Premium	Zepp AI / Zepp Coach	Personalized AI coaching	1
COROS	Free	COROS AI / EvoLab	Standard performance analysis	2
COROS	Advanced	COROS AI	Advanced training/performance analysis	3
Aaptiv / AI Fitness	Free/Trial	Aaptiv AI Coach	Standard AI fitness coaching	1
Aaptiv / AI Fitness	Paid	Aaptiv AI Coach	Personalized AI coaching	2
Nutrino / Medtronic	Free/Integrated	Nutrino FoodPrint	AI-based nutrition analysis	3
Nutrino / Medtronic	Advanced/Enterprise	Nutrino FoodPrint	Personalized nutrition recommendations	1
KitchinAI	Free	KitchinAI proprietary AI	Menu analysis / nutrition assessment	2
KitchinAI	Free	KitchinAI proprietary AI	Personalized menu analysis / health-aware assessment	3

https://doi.org/10.3390/nu18183106
